# Peri-Implantitis Regenerative Therapy: A Review

**DOI:** 10.3390/biology10080773

**Published:** 2021-08-13

**Authors:** Lorenzo Mordini, Ningyuan Sun, Naiwen Chang, John-Paul De Guzman, Luigi Generali, Ugo Consolo

**Affiliations:** 1 Department of Periodontology, Tufts University School of Dental Medicine, Boston, MA 02111, USA; ningyuan.sun@tufts.edu (N.S.); nai-wen.chang@tufts.edu (N.C.); johnpaul.deguzman@tufts.edu (J.-P.D.G.); 2 Department of Surgery, Medicine, Dentistry and Morphological Sciences with Transplant Surgery, Oncology and Regenerative Medicine Relevance (CHIMOMO), University of Modena and Reggio Emilia, 41124 Modena, Italy; luigi.generali@unimore.it (L.G.); ugo.consolo@unimore.it (U.C.)

**Keywords:** peri-implant disease, peri-implant mucositis, peri-implantitis, re-osseointegration, regenerative therapy

## Abstract

**Simple Summary:**

Regenerative therapies are one of the options to treat peri-implantitis diseases that cause peri-implant bone loss. This review reports classic and current literature to describe the available knowledge on regenerative peri-implant techniques.

**Abstract:**

The surgical techniques available to clinicians to treat peri-implant diseases can be divided into resective and regenerative. Peri-implant diseases are inflammatory conditions affecting the soft and hard tissues around dental implants. Despite the large number of investigations aimed at identifying the best approach to treat these conditions, there is still no universally recognized protocol to solve these complications successfully and predictably. This review will focus on the regenerative treatment of peri-implant osseous defects in order to provide some evidence that can aid clinicians in the approach to peri-implant disease treatment.

## 1. Introduction

Due to the increasing number of dental implants placed every day in clinical practice, the biological complications related to these treatments are increasing. These complications range from inflammation and bleeding upon probing (BOP) to severe peri-implant bone resorption and implant failure [1]. Despite many investigations aimed at identifying the best approach to treat these conditions, there is still no universally recognized protocol to solve these complications successfully and predictably. The techniques available for the clinicians can be divided into non-surgical and surgical. Among the surgical options, the two main approaches are resective and regenerative treatments.

Several methods have been explored to determine the most predictable and successful treatment protocol for arresting or reversing the loss of peri-implant bone. These methods included non-surgical, resective, and regenerative treatments along with various methods of adjunctive surface decontamination [2]. Generally speaking, if the peri-implant defect does not have bone walls or has a supra-bony component, the resective approach is usually preferred. The focus of this review will be the regenerative treatment of peri-implant osseous defects. The goals of this article will be to answer questions to assist clinicians pursuing peri-implant disease treatment: is any product superior to the other? Should a membrane be added to the graft? Is any method of decontamination superior? The authors reviewed recent studies on peri-implant regeneration and their outcomes, with background studies that led to the current knowledge of materials and techniques. Although this paper does not represent a systematic review, an effort was made to include as many scientific references on peri-implantitis surgical regenerative treatments. An electronic search in the database PubMed (National Library of Medicine) was performed. English was the only language included and the search was concluded in February 2021. The list of references was screened in order to exclude papers that did not include regenerative treatment of peri-implantitis. The following search terms were employed: ((peri-implant disease OR periimplant disease OR peri-implantitis OR peri implantitis) AND (guided bone regeneration OR regeneration OR re-osseointegration)) and (re-osseointegration OR reosseoitegration). The articles obtained from the electronic searches (857) were included only if they mentioned regenerative techniques on the surgical treatment of peri-implantitis. First, titles, and abstracts were assessed and those fulfilling the eligibility criteria were included. Secondly, the full texts were obtained and evaluated by the authors. References of systematic reviews was also obtained and reviewed, in case they were not present in the initial search.

## 2. Osseointegration and Re-Osseointegration

One important factor in evaluating implant placement success is osseointegration [3,4,5]. Implant osseointegration was defined as the “direct structural and functional connection between ordered living bone and the surface of an implant, without intervening fibrous tissue” [6]. Like osteogenesis, osseointegration is also comprised of bone formation and remodeling. In a rat study, Guglielmotti et al. found de novo woven bone around the implant six days after placement. Lamellar bone becomes present at 12–13 days, which signifies bone maturation. With additional bone formation occurring, the osseointegration process was still observed over two months post-implantation [7]. Implant osseointegration is affected by several factors. The characteristics of implant/tissue interface are considered as one of the critical local factors for osseointegration. Besides the implant surface treatment, the utilization of graft materials, platelet-rich plasma (PRP), and collagen materials can be considered local factors that may improve osseointegration [8]. Systemically, there are factors that might impair osseointegration (anemia, liver alteration, diabetes), as well as some systemic drug administration, may impair osseointegration (radiation therapy) or improve the osteogenic response (dexamethasone application) [9,10,11,12]. Systemic factors like blood cholesterol, glucose, and vitamin D levels may also contribute to bone healing around the implants [13].

The classic method for verifying peri-implant osseointegration is light microscopy analysis of undecalcified sections of the bone to implant connection [14]. This method involves a qualitative and quantitative analysis. The qualitative analysis focuses on the identification and description of different tissues, specifically mineralized and unmineralized fibrous connective tissues. The quantitative analysis is defined by histomorphometry as it describes the characteristics at the bone-implant junction and in the surrounding peri-implant bone [15]. The standard parameters analyzed are the bone area fraction occupancy, bone-to-implant contact (BIC), and the mineral apposition rates. These parameter outcomes are related to the quality of the histological specimens and to the recognition of artifacts that can falsify the true nature of the bone–implant interface [16]. BIC has always been measured either histomorphometrically or radiographically.

If the disease affects peri-implant bone, causing its resorption, the regenerative therapy is aimed at repairing and restoring the missing peri-implant structures through re-osseointegration. Some authors defined this term as the establishment of de novo osteogenesis and osseointegration [17], especially after peri-implant bone loss and over a previously bacterial contaminated implant surface [2]. Previous studies have shown several factors affecting the treatment of peri-implantitis and re-osseointegration outcomes; the most important were the efficacy of biofilm removal, quality of implant surface decontamination and conditioning, successful defect sites correction for adequate oral hygiene maintenance, effective plaque control, and use of grafts with growth factors for tissue regeneration [18]. In general, achieving re-osseointegration and long-term stability are considered the ultimate goals of peri-implantitis treatment [19].

## 3. Peri Implant Diseases

**Figure 1:**
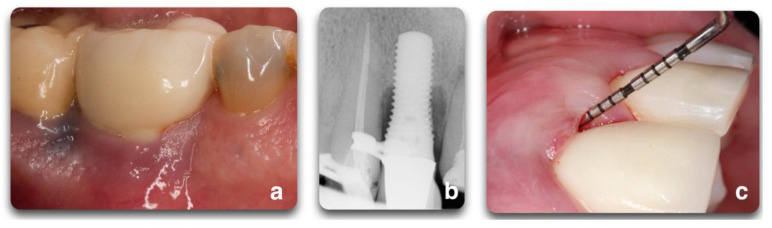
Representation of peri-implant clinical parameters that, associated, can lead to a diagnosis of peri-implantitis. In the sequence above, BOP/Suppuration (**a**), radiographic bone level ≥ 3 mm (**b**) in combination with PD ≥ 6 mm (**c**).

Implant status ranges from clinical health to implant failure and loss. In particular, the implants are classified as health, peri-implant mucositis, and peri-implantitis. The review by Araujo and Lindhe in the 2017 World Workshop [20] described the healthy peri-implant mucosa as comprised of either a keratinized (masticatory mucosa) or non-keratinized epithelium (lining mucosa) with underlying connective tissue. The clinical criteria available to assess and diagnose implant conditions are the same used to assess and diagnose periodontal conditions around teeth: probing depth (PD), bleeding on probing (BOP), suppuration (SUP), and radiographs. These can identify an inflammatory status and periodontal/peri-implant bone loss. The same authors stated that peri-implant health requires the absence of clinical signs of inflammation such as erythema, swelling, and bleeding on probing (BOP). Radiographic evidence of crestal bone changes around implants is important when differentiating peri-implant health from disease. When inflammatory signs appear (BOP, erythema, soft tissue swelling), a diagnosis of peri-implant mucositis (PIM) can be done. When PIM is associated with the progressive loss of supporting peri-implant bone, the diagnosis of peri-implantitis (PI) is established [1]. It is generally accepted that 0.5 to 2 mm of crestal bone loss during healing is considered physiological bone remodeling following implant installation and initial loading [21]. However, any additional radiographic evidence of bone loss more than 2mm after the placement of the prosthetic supra-structure, would suggest PI [21]. Although the conversion from an inflammatory process identified as PIM to PI is not well understood, it is generally agreed that both diseases share the same infectious etiology through the development of biofilm [1]. If the lesion is left untreated, the inflammatory process can lead to progressive peri-implant bone loss and implant failure. In case of an absence of documentation from the time of implant placement to the time of disease manifestation, radiographic bone level ≥ 3 mm in combination with BOP and PD ≥ 6 mm is indicative of peri-implantitis [21] (Figure 1).

## 4. Peri-Implantitis Treatment Factors

The goal of peri-implantitis treatments is re-osseointegration or bone fill of the osseous defect to provide support to peri-implant soft tissue and thereby improve esthetic outcomes [2,22]; surgical regenerative approaches are indicated to achieve this goal. Nevertheless, these techniques are not always applicable due to varying defect morphologies and progressively advancing stages of the disease.

**Figure 2:**
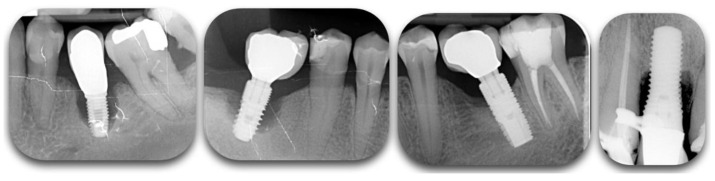
Radiographic films showing different degrees of peri-implant bone loss. The change of contrast can make the defect difficult to categorize.

Re-osseointegration or regeneration, by definition, is an event that can only be assessed histologically in experimental models [2,22]. The efficacy of peri-implantitis treatment can vary depending on the outcome variables [22]. In fact, clinical protocols are limited to bone level assessments by radiographs and clinical variables (BOP, PD, REC), while experimental protocols also include histological evaluations regarding inflammation resolution and osseous defect repair [22]. Clinicians cannot truly assess re-osseointegration on their patients unless a histological specimen is harvested and analyzed. Radiographic investigations are commonly used as the other non-invasive tool to assess bone changes after therapy [23], although questions regarding their reliability have been reported [16,24] (Figure 2). The most accurate solution to identify defect configuration is by direct access during the surgical intervention [25].

### 4.1. Peri-Implant Defect Configuration

**Figure 3:**
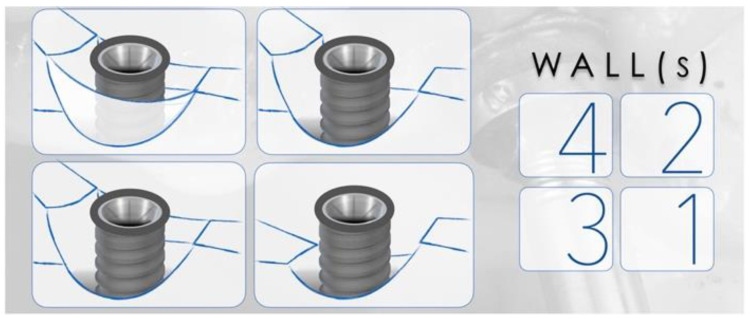
Representation of peri-implant defects according to the number of walls surrounding the lesion. The right panel corresponds to the number of walls present in the representations. The higher the number of walls, the higher is the positive outcomes of regenerative therapy.

One of the most important factors related to the success of peri-implantitis regenerative procedures is the peri-implant bone defect configuration. These procedures are not aimed at addressing disease resolution but are an attempt to fill the defect created by the disease. The feasibility of this goal has been shown to be closely associated with the configuration of the peri-implant defect and the number of walls surrounding the lesion (Figure 3).

**Figure 4:**
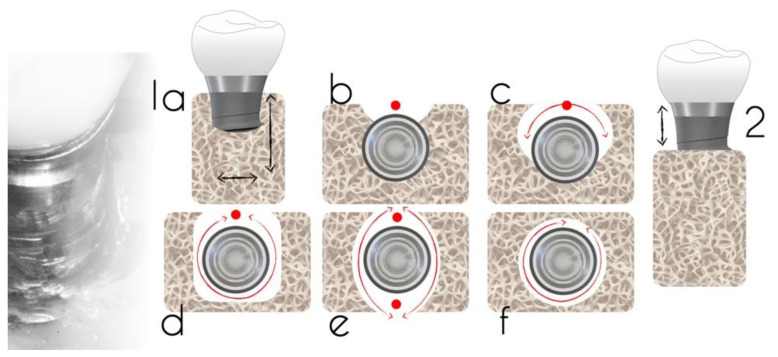
Defect classification according to an intra-operative assessment (adapted from Schwarz et al. [27]). Class 1a—facial view (**a**); Class 1a—occlusal view (**b**); Class 1b—buccal dehiscence + semicircular bone resorption to the middle of the implant body (**c**); Class 1c—buccal dehiscence + circular bone resorption with the lingual wall (**d**); Class 1d—circular bone resorption with lack of buccal and lingual walls (**e**); Class 1e—circular bone resorption with the presence of buccal and lingual wall; (**f**) Class 2: supra-crestal bone resorption.

**Figure 5:**
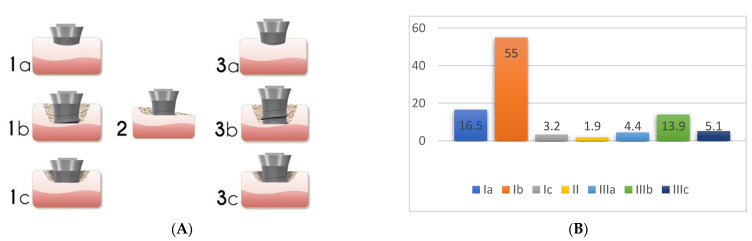
Peri-implant bone defect classification according to Monje et al. [23] (Adapted from original paper) (**A**). The figure is a representation of: Class I Infra-osseous defects, Class II Supra-crestal/Horizontal defects, and Class III Combined defects. Sub-classifications a (buccal dehiscence), b (2–3 wall defects), and c (circumferential configurations). (**B**) Prevalence of the different defect morphology types according to Monje et al. [23].

Studies have described a relationship between the peri-implant defect morphology and the clinical success of peri-implantitis therapies [23,25,26]. Therefore, careful consideration of the defect morphology must be made when selecting a peri-implantitis intervention. In 2007, Schwarz et al. [27] proposed a classification of peri-implantitis defects verified through intra-surgical findings in humans and animals. The classification was based on intrabony features and horizontal bone loss, describing the absence of buccal and/or lingual walls, circumferential, and supra- or sub-crestal patterns (Figure 4). In 2019, Monje et al. [23] updated this classification by adding combined defects (Figure 5A).

Prevalence data for the defect configurations seems to vary among studies. Schwarz et al., found circumferential defects to be the most prevalent (55.3%) [27]. In contrast, Garcia-Garcia et al. [28] found about 30% of defects to exhibit a circumferential configuration, while 25% included a buccal dehiscence combined with a circumferential defect. Aghazadeh et al. [25], on the other hand, found two-wall defects to be the most prevalent. Roccuzzo et al. [29] also introduced dissimilar data when exhibiting that one-third of cases (35.7%) had a semi-circumferential component combined with a buccal dehiscence. In a more recent study, Monje et al. [23] described three-wall defects as the most prevalent, followed by buccal dehiscence defects (Figure 5B).

It has been noted that the inconsistencies in these findings may be attributed to anatomical variations at implant placement. Schwarz et al. [27] described that the alveolar ridge width played a role in the number of bony walls formed in the future peri-implantitis defect. Moreover, the studies varied considerably in their anatomical location. For instance, most of the implants analyzed by Schwarz et al. were placed in the posterior region while other studies evaluated implants placed in the anterior and premolar regions [25]. Nonetheless, identifying the defect morphology yielded the opportunity to evaluate the predictability of various peri-implantitis treatments.

**Figure 6:**
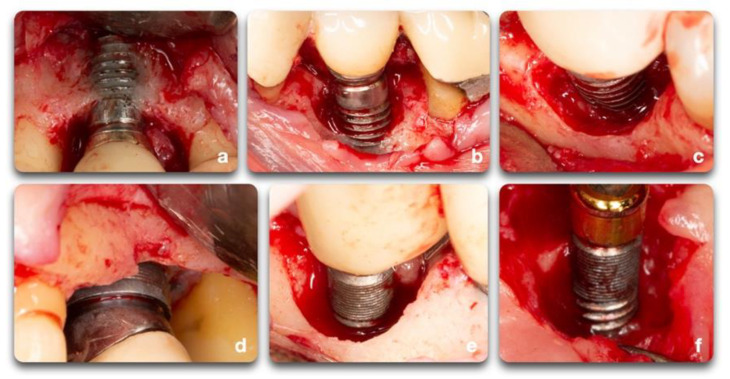
Clinical example of peri-implant bone defects; (**a**) facial dehiscence; (**b**,**c**) facial dehiscence and a semi-circumferential defect; (**d**) circumferential defect with a supra-crestal bone resorption; (**e**) circumferential defect; (**f**) semi-circumferential and supra-crestal bone defect.

Understanding the peri-implant defect morphology is important because of its potential for determining the likelihood of regeneration therapy success [25]. For situations in which regenerative procedures are unlikely to produce favorable results, resective surgeries may offer more clinical benefits. Of all the defect configurations, circumferential defects (Ie) achieved the highest reduction in PD and clinical attachment level (CAL) [26], while class Ib and Ic defects showed the poorest. Class Ib defects were the most prevalent in many of the studies. Therefore, it is more common to see peri-implantitis defect morphologies that are poorly responsive to reconstructive therapies (Figure 6).

### 4.2. Surface Decontamination

**Figure 7:**
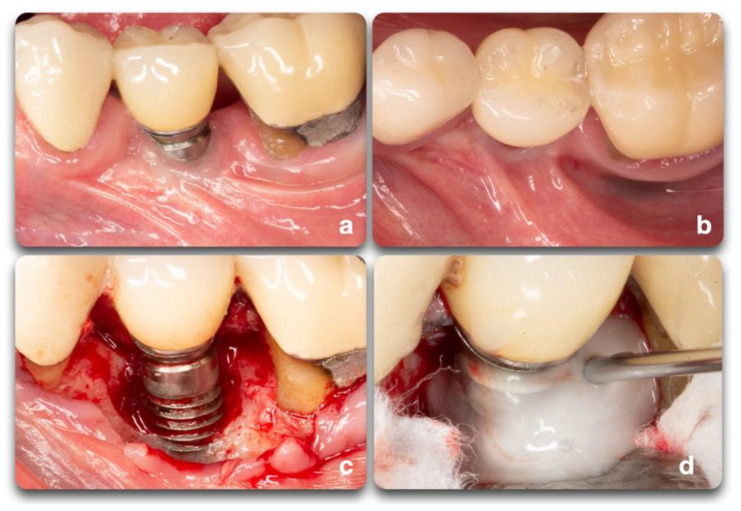
Clinical scenario representing a peri-implant defect affecting implant #20. A lack of keratinized mucosa (**a**) and frenum pull (**b**) could have played an important role in the defect shown (**c**). After mechanical debridement with curettes and titanium rotary brushes, the surface was treated with 24% EDTA gel to provide the chemical surface decontamination (**d**).

The implant surface holds great importance on the success and speed of osseointegration. Different implant brands are characterized by varying treatment surfaces and roughness [30]. The question on implant surface characteristics and healing following surgical therapy to treat peri-implantitis is not new. It is demonstrated that, as part of the regenerative procedure, implant decontamination is essential to obtain positive outcomes [31]. The consensus report from the 8th European Workshop on Periodontology [32] stated that implant surface decontamination is a critical component of surgical treatment. Implant decontamination is aimed at removing bacterial biofilm and resolving infection and inflammation, rendering the surface biocompatible and conducive to bone regeneration and possible re-osseointegration, or at least minimizing bacterial adhesion [2]. Various techniques have been proposed for implant surface decontamination after surgical exposure: mechanical, chemical, laser or photodynamic, or a combination of these [33] (Figure 7). The decontamination process presents multiple challenges. Besides the attempts to solve the infectious process, the implant threads and rough surfaces pose a significant obstacle to the mechanical cleansing that, if not optimal, can lead to the reestablishment of pathogenic microflora and persistence of pathology [33]. In advanced peri-implant defect lesions, surface decontamination alone will not adequately achieve bone regeneration. In these cases, filling the defect with graft materials and growth factors yields better outcomes. Investigations on surgical treatment for peri-implantitis range from in-vitro and animal studies to human clinical trials. Each of these fields provided important insight on outcomes and healing processes. Due to the limitations of each model, study design, length of treatment, materials, outcome measures, and the heterogeneity of data, it is difficult to compare outcome measurements [31,33,34,35,36].

#### 4.2.1. Pre-Clinical Studies

Pre-clinical experimental studies used a variety of methods to assess re-osseointegration following surgical therapy of peri-implantitis affecting implants with various surface characteristics [37,38,39]. Wetzel et al. [37] used reference points to indicate the most apical area of the peri-implant defect during surgery, while Persson et al. [38] utilized a fluorochrome marker following surgical therapy. In both studies, the amount of re-osseointegration on rough implants (sand-blasted, acid-etched) was superior to smooth, polished surfaces. Similar results were obtained by Namgoong et al. [39] who reported larger amounts of re-osseointegration at implants with sand-blasted, acid-etched/hydroxyapatite-coated surfaces than at implants with a turned/machined surface.

The results presented by these authors are in contrast with data reported by Almohandes et al. [22], who showed that re-osseointegration was significantly more frequent at smooth compared to rough surface implants (96% vs. 54%). The odds ratio for smooth implants to achieve re-osseointegration was ~25 compared to rough implants. The authors demonstrated that smooth surfaces showed a significantly higher radiographic bone level gain, enhanced resolution of peri-implantitis lesions, and a larger frequency of re-osseointegration sites.

#### 4.2.2. Human Clinical Studies

**Figure 8:**
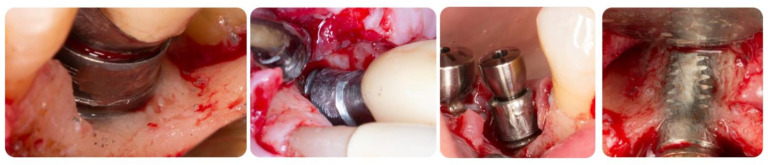
Intra-surgical view of peri-implant surfaces decontamination by means of implantoplasty. It is noticeable how difficult it is to completely remove titanium debris from the tissues, how it is not possible to completely reach the implant surface in narrow defects, and how much implant structure may have to be removed to accomplish smoothness. Narrower implants may risk fracture due to structural modification.

Implant surface characteristics were also identified to influence the results of peri-implantitis surgical therapies in clinical human studies. Roccuzzo et al. [29,40] prospectively evaluated a regenerative surgical treatment with a bovine-derived graft for peri-implantitis lesions on two different implant surfaces. A one-year follow-up resulted in clinical and radiographic improvements. The authors concluded that surface characteristics may have an impact on the clinical outcome after regeneration techniques and that complete defect fill was not always predictable. The authors reported that healing was superior around sandblasted, large grit, acid-etched (SLA) test surface implants than those with a rough titanium-plasma spray (TPS) control surface. Aghazadeh et al. [25] treated two groups of subjects with autogenous bone or bovine-derived xenograft in conjunction with a collagen membrane. The decontamination protocol consisted of mechanical debridement (titanium instruments) and hydrogen peroxide (H_2_O_2_). After 1 year, the bovine-derived xenograft showed significantly better results for bone levels, BOP, plaque index (PI), and suppuration. Isehed et al. [41], in an RCT, demonstrated that surface treatment with Emdogain^®^ (EMD) combined with sodium chlorohydrate could switch the microbiota to Gram positive aerobic bacteria and lead to an increase in bone levels. Jepsen et al. [42] utilized titanium brushes and H_2_O_2_ prior to placing titanium granules within the defects. It was not possible to demonstrate significant clinical benefits, but only marginal bone gain. Roccuzzo et al. [43] chemically treated the implant diseased surfaces with 24% EDTA and 1% Chlorhexidine before grafting the sites with DBBM + 10% collagen. Both authors reported PD reduction, inflammation resolution, and radiographic bone fill. In a 4-year follow-up study, Schwarz et al. [44,45] compared the 48 and 84 months regenerative outcomes of two decontamination techniques: Er:YAG laser and plastic curettes with cotton pellets and sterile saline. They did not find a statistically significant difference in the two treatment modalities. In a case series, Nart et al. [46] performed implantoplasty and filled the defects with allografts mixed with tobramycin and vancomycin (50%–50%) and membranes. The results showed positive outcomes in terms of radiographic bone fill, PD reduction, and CAL gain after a 12 months period. In these last studies, the implants were treated with implantoplasty, which removed the surface properties of the fixtures (Table 1). It is debatable whether this procedure should be performed or not and conflicts exist among the literature [47,48] (Figure 8).

Current evidence in the literature regarding the different clinical decontamination protocols has shown that complete implant surface decontamination (mechanical and chemical) could not even be achieved in vitro; there is a large variation in the effectiveness of the various approaches depending on the type of implant surface [33,48]. Despite the positive results of these studies, decontamination techniques standardization and comprehensive evaluation for true efficacy [33]. There is no evidence of clinical, radiographic, or microbiological data to favor one specific decontamination method over another. Further clinical investigations are needed to determine the superiority of a decontamination method, if possible [33].

## 5. Regeneration Techniques & Materials

The validation of reconstructive surgical therapy in peri-implantitis should be investigated in pre-clinical trials before applying it to human studies since evidence of re-osseointegration can only be confirmed histologically [2]. Due to the ethical considerations on humans, animal studies have been utilized to demonstrate successful bone regeneration around a previously affected implant [22,51,52,53,54]. Many parameters need to be controlled, such as bone substitute materials, membranes, implant surface characteristics, and their decontamination to understand their different roles in re-osseointegration. The scientific literature still lacks strong consensus demonstrating the absolute benefit of bone substitute materials in successfully repairing and/or regenerating peri-implant bone defects [22].

### 5.1. Animal Studies

Schwarz et al. (2007) [27] demonstrated a similarity between naturally occurring peri-implantitis in humans and ligature-induced peri-implantitis in beagle dogs. The results showed comparable configurations and dimensions of the defects, which concluded that the dog model could be a valuable representation of human defects. In a systematic review on re-osseointegration after surgical treatment of peri-implantitis, Madi et al. [19] and Renvert et al. [2] investigated the success rate of different protocols. After the surgical treatment of ligature-induced peri-implantitis, numerous methods to promote re-osseointegration were studied, such as regeneration with or without membranes and with or without bone grafts, laser treatment, and growth factors. Favorable results were observed in the studies that used a combination of bone grafts in guided bone regeneration therapy [19]. Re-osseointegration was achieved in some reports but it was highly variable. No methods were found to predictably resolve the peri-implant defects [2]. For instance, Schou et al. [51,52,53] performed a series of experiments on monkeys, with surgical treatment of ligature-induced peri-implantitis. Non-resorbable membranes (Expanded Polytetrafluoroethylene or ePTFE) with autogenous bone graft (ABG) or xenograft (Bio-Oss) were employed; At the histologic analysis, both combinations resulted in re-osseointegration (36% in the Bio-Oss group and 45% in the autogenous). Almohandes et al., 2019 [22], investigated the effect of bone substitute materials on hard and soft tissue healing in regenerative surgical therapy of dog ligature-induced peri-implantitis affecting implants with rough and smooth surfaces. The mean radiographic bone level (RBL) gain was significantly larger in the smooth implants (1.32 ± 0.69 vs. 0.27 ± 1.76 mm), showing more favorable outcomes in relation to the surface of the implant rather than the treatment rendered. In fact, in the rough implant group, the best radiographic result was achieved by controls, where no grafting material was added. Moreover, the additional use of a collagen membrane did not seem to impart additional benefits on the outcomes. The histologic analysis confirmed that smooth implants consistently showed better results with higher bone levels regardless of the regeneration technique. This trend was almost halved at the moderately rough sites. The issue seemed to depend on the quality and extent of implant decontamination, which is more challenging on rough surfaces [22]. These findings are consistent with results presented in a dog study by Ramos et al. [54], who reported that the use of the bone filler material did not improve results regarding re-osseointegration and bone level gain.

### 5.2. Adjunctive Therapies

Some authors have investigated the use of growth factors or lasers to understand the potential additive effect on re-osseointegration. You et al. [55] used ABG with or without Platelet Enriched Fibrin (PRF) after decontaminating the diseased implants with alternating gauze soaked in 0.1% chlorhexidine and saline. Re-osseointegration (~50%) was identified in the treatment group featuring ABG + PRF without membranes. Park et al. [56] applied three treatment modalities after surgical defect exposure: Hydroxyapatite (HA) particles and collagen gel (control), HA with collagen gel containing autologous periodontal ligament stem cells (PDLSCs), and HA particles with collagen gel containing BMP-2–expressing autologous PDLSCs. Despite no significant difference between groups regarding BIC, the histological specimens showed a significantly higher amount of re-osseointegration for BMP2/PDLSC group (2.1 mm) and 61% of the defect were regenerated. Shibli et al. [57] tested a photosensitization technique, as the combination of low-level diode laser + toluidine blue O, on 4 different implant surfaces without the use of biomaterials but covered with ePTFE membranes. The authors concluded that photosensitization could provide significant bone fill with re-osseointegration. Machtei et al. [58] evaluated the effect of a bone substitute material, beta tri-calcium phosphate (β-TCP), with or without endothelial progenitor cells (EPC). It was reported that the combination of β-TCP and EPC enhanced bone formation after surgical therapy, while differences between the β-TCP group and controls were small.

The benefits derived from using bone grafts and membranes were not always significant. In other words, the use of bone substitute materials was not essential for bone level gain in radiographs, resolution of peri-implantitis lesions, and occurrence of re-osseointegration. Thus, experimental studies investigating the benefit of bone substitute materials in the management of peri-implantitis-associated osseous defects are few and do not provide evidence to support for use of those graft materials. Bone substitute materials do not provide obvious advantages in achieving bone fill or re-osseointegration, even though interpretation must be made with care and considering the specific nature of the experimental model [2,22]. The osseous defect that occurs in the dog mandible following experimental peri-implantitis often demonstrates a contained, symmetric morphology with well-preserved bone walls. Bone healing that occurs after surgical therapy is favored by the contained morphology of the bone defect more than the potential benefit of placing a bone substitute material [22].

### 5.3. Studies on Humans

Regenerative procedures around teeth imply reconstitution of the lost attachment apparatus composed of bone, cementum, connective tissue, and periodontal ligament [59]. If translated in the dental implant realm, bone regeneration and re-osseointegration are the sole objective therapeutic goal in specific peri-implant bony defects on functioning implants [2,3]. Daugela et al. [60] performed a systematic review of the literature to identify the most effective therapeutic predictable option on the surgical regenerative treatment of peri-implantitis. The review revealed that the weighted mean Radiographic Bone Level (RBL) fill was close to 2 mm, PD reduction was 2.78 mm and BOP reduced by more than 50%. Defect fill, in studies using and not using barrier membranes for graft coverage, were 1.86 mm and 2.12 mm, respectively. High heterogeneity among the studies regarding defect morphology, surgical protocols, and selection of biomaterials was detected. The results showed an improvement of clinical scenarios after the surgical regenerative approach of peri-implantitis; however, the authors could not find scientific evidence regarding the superiority of the regenerative versus non-regenerative surgical treatment. They also concluded that the use of a barrier membrane or submerged healing did not seem to be mandatory for a successful outcome. A contrasting conclusion was derived from a systematic review from the AAP Task Force in 2014 stating that although the evidence was limited, the use of grafting material and barrier membranes may contribute to a better reduction of PD and defect fill [61]. A consensus report from Khoury et al. [31] acknowledged that surgical regenerative peri-implantitis therapy improved clinical and radiographic treatment outcomes compared with the baseline with up to 10 years of follow-up. However, the authors did not find evidence to support the superiority of a specific material, product, or membrane in terms of long-term clinical benefits. The surgical approach to treat peri-implantitis may be justified when the non-surgical approach failed, evidenced by the persistence of BOP and suppuration. Ramanauskaite et al. [34] reviewed the literature to identify the difference in clinical and radiographic outcomes between regenerative and non-augmentative surgical therapies. Regenerative peri-implantitis therapy demonstrated significant improvements in BOP and PD values compared to the baseline. In particular, the mean BOP reduction ranged from 25.9% to ~90% and 91% in a 1- to 7-year period, and the mean PD reduction ranged from 0.74 to 5.4 mm. The mean radiographic bone fill ranged between 57% and 93.3%. Furthermore, the radiographic reduction of the intrabony defect height varied from 0.2 to 2.8 mm and up to 3.70 and 3.77 mm. A variety of bone grafting materials were applied (autogenous bone, alloplasts, xenograft, and titanium granules) with and without resorbable or non-resorbable membranes [34]. Xenografts demonstrated significantly higher radiographic bone level gain, compared to ABG, in the short term. The reasons could be attributed to the greater radio-opacity of these materials compared with ABG.

In a recent systematic review by Aljohani et al. [62] the regenerative treatments were compared according to PD, BOP, and RBL. The materials evaluated included autogenous bone compared to bovine-derived xenografts with a resorbable collagen membrane, porous titanium granules without membranes, and autogenous bone mineral with a collagen membrane. Lastly, the detoxification methods included in the review were 3% H_2_O_2_ and saline, 24% EDTA gel and saline, implantoplasty and Er:YAG laser, and sterile saline only. All the interventions demonstrated a significant decrease in PD when compared to the baseline (before the intervention) PD measurements. However, the difference in mean PD among all the studies was not statistically significant. Aghazadeh et al. [25] achieved the highest mean reduction in PD (3.1 mm) with the use of bovine-derived xenograft and a collagen membrane. The lowest reduction in PD (1.2 mm) was observed in patients treated with implantoplasty and a saline rinse. This decontamination method reduced BOP by 85.2%. When evaluating the RBL, most studies showed an increase compared to baseline. However, this parameter also failed to exhibit statistical significance in all groups.

**Figure 9:**
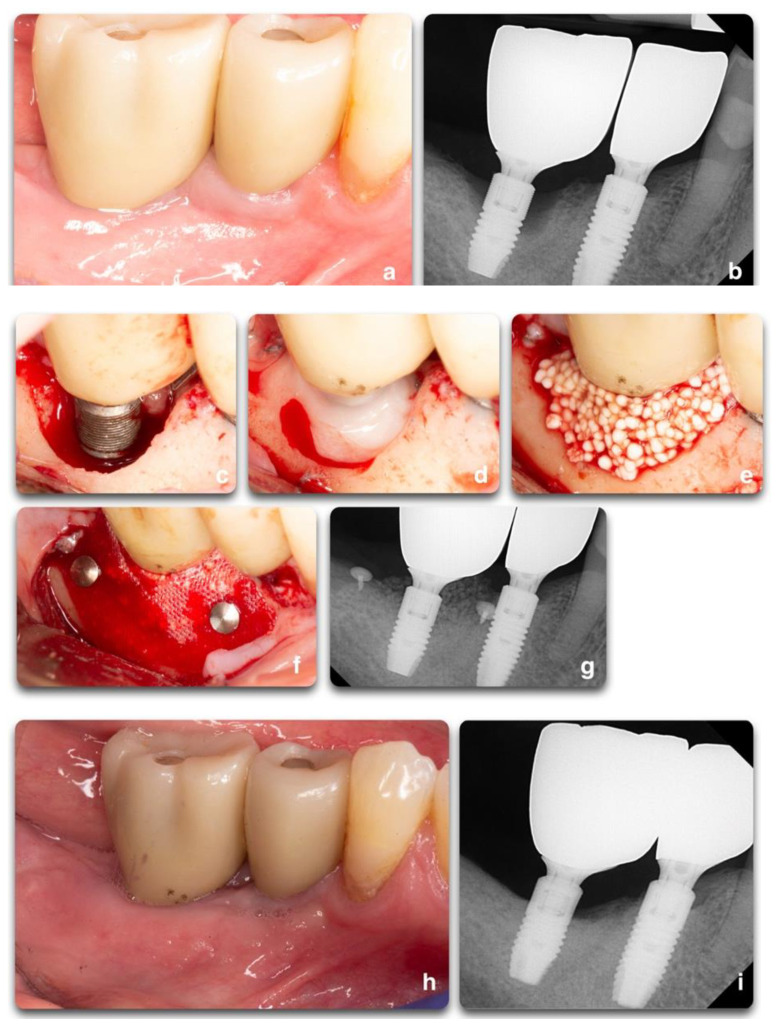
Regenerative peri-implant therapy for implant area #31. (**a**,**b**) Pre-operative clinical and radiographic presentation. BoP, SUP, PD 10mm. (**c**) Defect configuration after the elevation of the full-thickness flap on implant #31. (**d**) Implant surface decontamination with rotating titanium brushes, saline and chlorhexidine rinse, and the application of PrefGel^®^ 24% EDTA (2021 Institut Straumann AG^®^). (**e**,**f**) Bone grafting application (GUIDOR^®^ easy-graft^®^ CLASSIC Alloplastic Bone Grafting System © © Copyright Collagen Matrix, Inc.—Allendale, NJ, USA), covered by the GUIDOR^®^ matrix barrier membrane, and stabilized by two titanium tacks. (**g**) Radiograph showing the grafting material at the time of surgery completion. (**h**,**i**) 13 months post-operative clinical and radiographic outcomes. Partial fill of the intra-bony defect with a residual supra-crestal defect.

**Figure 10:**
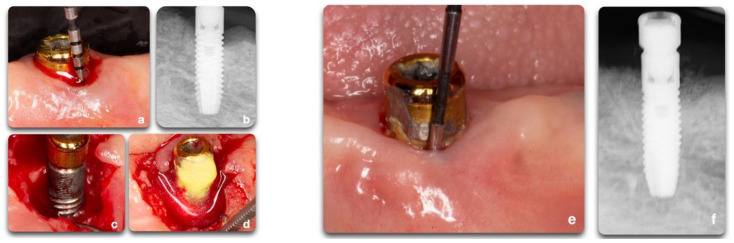
Regenerative peri-implant therapy for implant area #22. (**a**,**b**) Pre-operative clinical and radiographic presentation. BoP, SUP, PD > 5 mm. (**c**) Defect configuration after the elevation of the full-thickness flap. (**d**) Decontamination with rotating titanium brushes, saline, and chlorhexidine rinse, and 2 min of tetracycline hydrochloride soluble powder applied to the implant surface. Bone grafting application and flap closure around existing locator abutment (LOCATOR Astra Tech EV Overdenture Abutment © Dentsply Sirona); (**e**,**f**) 12 months post-operative clinical and radiographic outcomes. To be noted is the partial fill of the intra-bony defect with a residual supra-crestal fixture.

By using porous titanium granules, Jepsen et al. [42] reported the greatest mean defect fill when compared to other interventions. Overall, the five studies included demonstrated improvements in clinical conditions when compared to baseline. Nevertheless, there was no statistically significant difference in PD, BOP, or RBL when comparing the studies to each other. The authors believe that the reduction in BOP was the outcome of the normal healing process after the surgical treatment rather than the materials used or decontamination methods utilized. The authors further suggested that the porous titanium granules may be the best bone substitute to achieve the greatest RBL. Xenograft would be the next best, and autogenous bone would come after. A limitation of evaluating RBL is that it does not indicate complete re-osseointegration. Therefore, because the autogenous bone is less radiopaque than titanium granules, it may not appear as having comparable radiographic bone levels. Two examples of regeneration using bone grafts are shown in Figure 9 and Figure 10.

## 6. Treatment Outcomes

### 6.1. Success of Regenerative Therapy

The success of regenerative therapy varies from study to study. The use of composite outcomes for treatment success is not standardized and varies among different studies [34,35]. Various authors reported different treatment outcome goals when defining treatment success (Table 2). A recent review [34] showed that, depending on the criteria used, treatment success varied between 11% and 38.5% (implant level in a 1-year period), and between 14.3% and 66.7% (implant level), and 60% (patient level), in a 5–7 years follow-up. A systematic review [63] from Sahrmann investigated the available literature for regenerative treatment of peri-implantitis using bone grafts and membranes. Qualitative measures showed ~10% of complete radiographic bone fill, 85.5% of incomplete defect resolution, and no bone fill in 4% of the cases. It is noted that a high heterogeneity among disinfection protocols and biologic materials used was noticed. This limitation made a meta-analysis impossible to be achieved. The clinical outcomes of surgical regenerative therapy were reported to be influenced by the implant surface characteristics [29] as well as by the peri-implant defect configuration. For instance, moderately rough or smooth surface implants seemed to outperform rough surface implants in terms of clinical treatment outcomes; furthermore, circumferential-type defects responded better to therapy compared to dehiscence-type defects [2,24,27,36].

### 6.2. Time Stability of Therapy

A systematic review from Heinz-Mayfield [35] investigated the treatment of peri-implantitis by defining the therapy successful (implant survival) if PD < 5 mm and no progressive bone loss 12 months after treatment. The studies varied in their decontamination methods, grafting materials, membrane usage, and implant surfaces. In a 12-month follow-up study, Roccuzzo et al. [40] found a mean radiographic bone gain of 1.7 mm and an incomplete defect fill in 75% of implants. Two implants were lost after developing suppuration. Wiltfang et al. [65], in a 12-month follow-up study, employed implantoplasty and a mixture of autogenous bone and demineralized xenogenic bone graft with growth factors and systemic antibiotics. The investigators reported an average reduction in PD (4 mm), in BOP (from 61% of implants to 25%), a mean gain in radiographic bone height of 3.5 mm, and an increased recession of 2 mm. Overall, one implant was lost due to mobility. Froum et al. [66] investigated the use of enamel matrix derivatives, bone graft mixed with PDGF, and a collagen membrane or subepithelial connective tissue graft. With a 3–7.5-year follow-up, researchers reported a mean PD reduction of 5.3 mm and radiographic bone gain of 3.4 mm. No implants exhibited recession, and no implants were lost. Haas et al. [67] investigated the use of penicillin and photodynamic therapy with autogenous bone and ePTFE membranes. Researchers reported a mean radiographic bone gain of 2 mm and the loss of two implants. Roos-Jansaker et al. [50] investigated the submerged approach using bone substitutes and resorbable membranes. At the 12-month follow-up period, implants exhibited a radiographic bone fill of 2.3 mm, a mean PD reduction of 4.2 mm, and BOP reduction from 75% to 13%. Schwarz et al. [26] studied the regenerative potential of the non-submerged healing using xenografts with a collagen membrane. The 12-month follow-up demonstrated PD reduction from 6.9 mm to 2.0 mm. In a 3-year follow-up study, Behneke et al. [68] used air-powder abrasives, autografts, and systemic metronidazole to treat peri-implantitis. The researchers reported no implant losses, a mean PD reduction of 3.1 mm, and a median marginal bone gain of 4 mm. These favorable outcomes were maintained for up to 3 years (Table 3). In a systematic review evaluating regenerative treatments by Heinz-Mayfield et al. [35], it was not possible to advocate one specific treatment as being more successful in achieving regeneration than the others due to the great heterogeneity among studies. Regardless, certain aspects of therapies seem to influence treatment outcomes positively. The beneficial factors found in the pretreatment phase include oral hygiene instruction and smoking cessation, prosthesis adjustments, and nonsurgical debridement. In terms of surgical access, the use of full-thickness mucoperiosteal flaps and the use of bone grafts or substitutes seem to be associated with improved treatment outcomes. Postoperative protocols seemed to positively influence treatment outcomes when antibiotics were used and when chlorhexidine rinse was included in the postoperative management. Lastly, maintenance care seemed to improve treatment outcomes when a 3- to 6-month interval was utilized.

## 7. Discussion

Animal research has demonstrated that it is possible to regenerate bone to achieve re-osseointegration on a previously infected implant surface [22,37,38,39,55,56,57,69,70,71]. These studies including histological evidence covered different methods of implant surface decontamination such as mechanical, chemical, and adjunctive (i.e., lasers). These pre-clinical studies tested regenerative procedures using membranes, bone grafting materials, and biologic factors on different surgical protocols. Multiple studies also investigated the effect of different implant surface decontamination techniques prior to regenerative procedures on the potential for re-osseointegration. Promising results were observed in the studies that used a combination of bone grafts and membranes even if others did not confirm the same outcomes. The multitude of techniques and related outcomes measures differed among authors. Presently, peri-implantitis treatment is not predictable and cannot be distinguished as a single effective protocol. The heterogeneity was high due to different protocol designs and animal models used, defect size and shape the number, location and kind of implants placed, the implant surface, the time of healing, peri-implantitis induction, treatment length, and timing of the animal sacrifices. Also, many protocols were used regarding pre-surgical plaque reduction, oral hygiene measures, and definitions for outcome measurements. Some studies failed to report some of the aforementioned information. As a result, it is difficult to reach an overall definitive conclusion.

Many studies agree that surgical treatment seems to be the most predictable treatment option when an adequate chemical and mechanical implant surface decontamination is achieved. In summary, based on animal studies, re-osseointegration can be achieved on a previously infected and contaminated implant surface. Re-osseointegration was highly varied among and between studies and was unpredictable. It may be seen in pre-clinical-induced peri-implantitis defects following regeneration and may also be influenced by the implant surface properties.

Human studies do not allow any definitive conclusions. According to the current evidence, it seems possible to achieve some defect fill and disease resolution by means of regenerative techniques using different bone substitute grafts (autogenous, xenograft, allograft, and titanium granules), with or without the adjunctive use of barrier membranes [19,31,34,35,36]. These regenerative techniques should be considered in areas of high esthetic demand and when the defect morphology is suitable for a predictable outcome [23,26]. The initial intraosseous defect fill could be maintained over time if low plaque and bleeding scores could be controlled by effective oral hygiene and frequent maintenance [49]. As with every disease, prevention is the best form of treatment, and peri-implantitis is no exception. The scarcity of human histological evidence still makes it difficult to form generalizations about the efficiency of regenerative procedures and their potential for re-osseointegration [36]. A determining factor is the defect configuration for a predictable outcome following regenerative treatment. Yet, several experimental studies demonstrated that even where in circumferential defects, the amount of regeneration achieved is limited. It is important to inform the patient clearly about the possibility of a recession and subsequent exposure of the body of the implant. Unlike natural teeth, peri-implant lesions do not seem to respond predictably to either non-surgical or surgical treatments [36].

The main common postoperative complication of regenerative therapies seems to be membrane exposure when used [35,58]. The recent consensus on peri-implant diseases identified strong evidence that lack of oral hygiene, history of periodontitis, and smoking are risk indicators for peri-implantitis [1]. It is so advised that, following implant placement, patients should be included in a strict and regular maintenance schedule [72]. If peri-implantitis is already established, the proposed strategies and recommendations for its treatment are still considered empirical [36]. From the existing evidence, it seems that nonsurgical therapy is not completely effective, at least not in advanced cases [36]. The advantage of surgical techniques is the ability to achieve adequate access to degranulate the inflamed tissues effectively, to modify implant surfaces, to decontaminate the implant, to reduce PD, and, when indicated, to attempt regeneration.

## 8. Conclusions

Based on the current evidence in reconstructive peri-implant therapy, regenerative surgical techniques demonstrated improvement of peri-implant clinical and radiographic parameters. Yet, there is not enough evidence to identify a specific grafting material or membrane that would grant long-term clinical treatment benefits over the others. No specific surface decontamination treatment can be considered superior in terms of influencing the clinical outcomes of regenerative peri-implantitis therapy. One of the most important treatment factors is the peri-implant bone defect morphology, which demonstrated its influence on the final therapy outcomes. The initial intraosseous defect fill could be maintained over time if low plaque and bleeding scores are controlled by effective oral hygiene and frequent maintenance. Regenerative therapies should be applied in specific and selected clinical scenarios.

## Figures and Tables

**Table 1:** Implant surface decontamination and regenerative techniques material employed by the reported human studies. The kind of surgical healing (submerged and non-submerged) was reported as well as the antibiotic therapy prescribed to research subjects. OHI (Oral Hygiene Instructions); NST (Non-surgical Therapy); ID (Implant Debridement); NC (Neck Cleaning); GD (Gingival Debridement); d (Day); wk (Week), m (month); MD (Mechanical Debridement); IP (Implantoplasty); Ti (Titanium); SCRP (Scaling and Root Planing); OFS (Open Flap Surgery); PC (Plastic Currette); AB (Autogenous Bone); M (Membrane); RM (Resorbable membrane); NR (Non-resorbable Membrane); ABX (Antibiotic); NS (Non-Submerged), XENO (Xenograft), EMD (Emdogain); CHX (Clorhexidine); T (Test); C (Control); CTG (Connective Tissue Graft); BID (Twice a Day); SS (Stainless Steel); US (Ultra Sonic); EDTA (Ethylenediaminetetraacetic Acid). Adapted from Ramanauskaite et al. [34].

Author	Pre-Surgical Therapy	Implant Decontamination	Grafting Material	Membrane	Healing	Systemic Antibiotics
Roos-Jansaker et al. [49,50], 2007, 2014	NR	T: H_2_O_2_ (3 min)	Algae-derived XENO	Resorbable synthetic membrane	NS	Amoxicillin 375 mg × 3/d + metronidazole 400 mg × 2/d, 10 d after the surgery
Aghazadeh et al., 2012 [25]	OHI	T & C: MD + H_2_O_2_ (1 min)	XENO	RM	NS	Post-operative ABX Azithromycin 2 × 250 mg 1/d, 1 × 250 mg 2–4/d
			AB			
Isehed et al., 2016 [41]	OHI	T: US + Ti Instruments + Cotton Gauze (NaCl)	EMD (0.3 mL)	No M	NS	No
		C: US + Ti instruments + Cotton Gauze (NaCl)	OFS			
Schwartz et al., 2011, 2013, 2017 [47,48]	NST + OHI	T: Er:YAG Laser + IP	XENO	RM	NS	No
		C: OFS + PC + Cotton Pellets & NaCl + IP				
Roccuzzo et al., 2011, 2017 [29,40]	ID + OHI	T (SLA): PC + 24% EDTA + 1% CHX gel	XENO	No M	NS	1g of Amoxicillin + Clavulanic Acid BID, 6 d
		C (TPS): PC + 24% EDTA + 1% CHX Gel				
Roccuzzo et al., 2016 [43]	OHI + SCRP (Teeth) + NC (Implant)	Ti Curettes + Ti Brush + 24% EDTA + 1% CHX Gel	XENO + 10% Collagen	If no KT: Tuberosity CTG	NS	1 g of Amoxicillin + Clavulanic Acid × 2, 1 h before Surgery × 6 d
Nart et al., 2018 [46]	OHI + Supra- & Sub-GD (6 wk before Surgery)	SS Curette + IP + US Intrabony Debridement + 3% H_2_O_2_ (1 min) + NaCl	50% Allograft & Vancomycin + 50% Allograft Tobramycin	RM	NS	No

**Table 2:** The success of regenerative treatment reported by the listed authors. As it is noted, the criteria vary among studies, making the concept of treatment success difficult to standardize. PD (Probing Depth); BL (Bone Loss); BOP (Bleeding on Probing); SUP (Suppuration); RF (Radiographic Fill); DF (Defect Fill). Adapted from Ramanauskaite et al. [34].

Success of Peri-Implantitis Regenerative Treatments		
Author	Success Definition	Success Outcome
Jepsen et al., 2016 [42]	PD ≤ 4 mm, no BOP at 6 implant sites, no further BL	30% of implants
Schwarz et al., 2017 [45]	No BOP	Test: 4/6 patients Control: 5/9 patients Total: 9/15 patients (60%)
Roccuzzo et al., 2017 [29]	PD < 5 mm, no BOP or SUP, no further BL	Test: 7/12 (58.3%) implants Control: 2/14 (14.3%) implants
Aghazadeh et al., 2012 [25]	PD ≤ 5 mm, max 1 site with BOP, no SUP, no BL PD ≤ 5 mm, no BOP, no SUP, no BL	Test: 38.5% implants Control: 13.9% implants Test: 8 implants (20.5%) Control: 4 implants (11.1%)
Roos-Jansaker et al., 2014 [49]	RF ≥ 25%, independent of PD or BOP; RF ≥ 25%, PD ≤ 5 mm, independent of BOP RF ≥ 25%, PD ≤ 5 mm, BOP ≤ 1	66.7% (30/45) implants 62.2% (28/45) implants 51.1% (23/45) implants
Renvert et al., 2018 [64]	DF ≥ 1.0 mm, PD ≤ 5 mm, no BOP, no SUP	Control: 1/20 (5.0%) Test: 9/21 (42.9%)

**Table 3:** Studies that reported on the number of patients (Pt) treated, % therapy success, mean probing depth (PD) change, % of bleeding and/or suppuration on probing, radiographic bone levels at 12 months after treatment. Successful outcomes are defined as: implant survival with a mean PD < 5 mm and no progressive bone loss 12 months after treatment. AB: Autogenous Bone; T0: Baseline; m: month; wk: week; Pt: patient; im: implant. Adapted from Ramanauskaite et al. [34].

Authors	Study	Pt	% Success Outcome	% Sites BoP	12 m Mean PD	Baseline PD	Bone Change (Radiographic)	Comments
Haas 2000 [67]	Clinical study	17 (24 im)	-	-	-	-	2 ± 1.9 mm (36.4%) (9.5 m)	- Membrane exposure in all patients (~3 wk PO) - 2 im failed
Bennhke 2000 [68]	Prospective (AB graft)	17 (25 im)	-	-	3.3 mm (median reduction 3 y)	-	- Mean bone defect fill: 3.7 mm - Median defect depth reduction 6.9 mm (re-entry) - Bone repair: 90%.	im lost in 6 patients
Roos-Jaskaren et al., 2007 [50]	Comparative trial: bone + membrane	17	93 im	22	2.5 mm	5.4 mm	2 im lost, 1 thread bone	-
	Bone	19	89 im	25	2.2 mm	5.6 mm	1 im lost 2 threads, 3 im lost 1 thread	-
Roccuzzo 2011	Case series	26	85 Pt	36	4.3 mm	7.0 mm	1.7 mm mean bone gain (12 m)	4 Pt with TPS-im SUP
Wiltfang 2010	Case series	22	75 im	25	3.5 mm	7.5 mm	3.5 mm mean bone gain (12 m)	SUP 8% im, 1 patient lost 1 implant
Froum et al., 2012 [66]	Case series	38	84 Pt	18	3.0 mm *	8.3 mm *	3.4 mm mean bone gain (12 m)	6 Pt required 2–3 surgeries no im lost bone
Nart et al., 2018 [46]	Case series	13	-	70.6	4.23 ± 1.62 mm (mean reduction)	7.88 ± 1.22 mm	Intrabony defect: T0 (mm): 4.33 ± 1.62 mm, after 12 m: 0.56 ± 0.88 mm Bone defect fill: 86.99 ± 18.2%	-
Renvert et al., 2018 [21]	RCT; surgical debridement	20	5.0	65 Control	3.9	6.0	0.2 mm (12 m)	32.8% risk reduction in benefit of test
	Surgical debridement + bone substitute	21	42.9	52.4 Test	2.6	6.6	0.7 mm (12 m)	

* Indicate *p* < 0.05.

## Data Availability

Data were collected by previous published studies available in the scientific literature.
